# Ventilator-associated tracheobronchitis: an update

**DOI:** 10.5935/0103-507X.20190079

**Published:** 2019

**Authors:** Jorge Ibrain Figueira Salluh, Vicente Cés de Souza-Dantas, Ignacio Martin-Loeches, Thiago Costa Lisboa, Ligia Sarmet Cunha Farah Rabello, Nseir Saad, Pedro Póvoa

**Affiliations:** 1 Instituto D'Or de Pesquisa e Ensino - Rio de Janeiro (RJ), Brasil.; 2 Programa de Pós-Graduação em Clínica Médica, Universidade Federal do Rio de Janeiro - Rio de Janeiro (RJ), Brasil.; 3 Faculdade de Medicina, Universidade Federal do Rio de Janeiro - Rio de Janeiro, (RJ), Brasil.; 4 Multidisciplinary Intensive Care Research Organization, Department of Clinical Medicine, Trinity Centre for Health Sciences, St. James's University Hospital -Dublin, Ireland.; 5 CIBER Enfermedades Respiratorias, Critical Care Center, Sabadell Hospital, Corporación Sanitaria Universitaria Parc Taulí, Universitat Autonoma de Barcelona - Sabadell, Spain.; 6 Rede Institucional de Pesquisa e Inovação em Medicina Intensiva, Complexo Hospitalar Santa Casa de Misericórdia de Porto Alegre - Porto Alegre (RS), Brasil.; 7 Comitê do Departamento de Terapia Intensiva e Controle da Infecção, Hospital de Clínicas de Porto Alegre, Universidade Federal do Rio Grande do Sul - Porto Alegre (RS), Brasil.; 8 Critical Care Center, Centre Hospitalier Universitaire de Lille - Lille, France.; 9 School of Medicine, University of Lille - Lille, France.; 10 Unidade Polivalente de Terapia Intensiva, Centro Hospitalar de Lisboa Ocidental, São Francisco Xavier Hospital - Lisboa, Portugal.; 11 NOVA Escola Médica, CEDOC, New Universidade de Lisboa, Campo Mártires da Pátria - Lisboa, Portugal.

**Keywords:** Critical care, Mortality, Nosocomial infection, Pneumonia, Healthcare-associated pneumonia, Pneumonia, ventilator-associated, Ventilator-associated tracheobronchitis

## Abstract

Ventilator-associated lower respiratory tract infection is one of the most frequent complications in mechanically ventilated patients. Ventilator-associated tracheobronchitis has been considered a disease that does not warrant antibiotic treatment by the medical community for many years. In the last decade, several studies have shown that tracheobronchitis could be considered an intermediate process that leads to ventilator-associated pneumonia. Furthermore, ventilator-associated tracheobronchitis has a limited impact on overall mortality but shows a significant association with increased patient costs, length of stay, antibiotic use, and duration of mechanical ventilation. Although we still need clear evidence, especially concerning treatment modalities, the present study on ventilator-associated tracheobronchitis highlights that there are important impacts of including this condition in clinical management and epidemiological and infection surveillance.

## INTRODUCTION

Ventilator-associated lower respiratory tract infection (VA-LRTI) is one of the most frequent complications in mechanically ventilated patients.^(1,2)^ There has been a reduction in the ventilator-associated pneumonia (VAP) prevalence over the last two decades.^(3)^ This decrease has been associated with improvements in the knowledge of physiopathology and the implementation of adequate programs for prevention.^(3)^ In contrast, ventilator-associated tracheobronchitis (VAT), which is characterized by signs of respiratory infection without new radiographic infiltrates in patients who have been ventilated for at least 48 hours, has been neglected and considered a disease that does not warrant antibiotic treatment by the vast majority of the medical community for many years.^(4)^

In the last decade, several epidemiological studies have shown that VAT could be considered a precursor of VAP. Furthermore, although VAT has a limited impact on mortality, it shows a significant association with increased patient costs, length of stay (LOS), antibiotic use, and duration of mechanical ventilation (MV).^(4)^

Although there are no consensual gold standard definitions to diagnose these infectious complications, diagnoses usually include clinical, radiologic and microbiologic criteria.^(1,5)^ Because no diagnostic criteria for VAT are widely accepted, its existence is still questioned by some authors, and its prevalence can vary from almost 0% to 15% in patients who are mechanically ventilated.^(6,7)^

An improved understanding of VAT could have important implications for early diagnosis, initiation of antimicrobials, and prevention. However, the concept of VAT is controversial, unlike VAP, and several important factors remain unclear, such as its definition, degree of overlap with VAP, diagnostic criteria, and appropriate treatment regimens.^(4)^

### Diagnosis and outcomes

As previously stated, one important aspect of VAT is the definition for such entities (Table 1). After a careful review of published papers that defined VAT, we concluded that definition might be, at times, a pure jigsaw. Some definitions considered etiologies, thresholds, or systemic symptoms; however, a common feature is the lack of lung infiltrates in portable chest X-ray (CRX). This is obviously a good common denominator, but identifying this feature in daily clinical practice is not as easy as it seems. For instance, in a study published by Self et al including patients admitted to an emergency department in the United States (US), they found a high discordance between CRX and computed tomography (CT) for the detection of pulmonary opacities.^(7)^ The authors concluded that CRX had poor sensitivity and a poor positive predictive value for detecting a new infiltrates, leading to significant rates of the misdiagnosis of pneumonia. Considering these findings and extrapolating the results to mechanically ventilated patients, diagnosis becomes even more difficult.

**Table 1:** Ventilator-associated events

Ventilator-associated condition	Increase in daily minimum PEEP of ≥ 3cm/water or by FIO_2_ > 0.2 sustained for ≥ 2 days
Ventilator-associated tracheobronchitis	VAC
	**PLUS (At least 2)**
	Temperature of < 36ºC or > 38ºC
	**OR**
	Leukocyte count of ≤4000 or ≥12,000/mm^3^
	**OR**
	Cough
	**OR**
	New or increased production of sputum
	**OR**
	Rhonchi and wheezing
	**OR**
	Gram staining of endotracheal aspirate or bronchoalveolar lavage showing ≥ 25 neutrophils and ≤ 10 epithelial cells/field
	**PLUS**
	Absence of new or progressive pulmonary infiltrates
	**PLUS**
	ETA with ≥10^5^ CFU/mm^3^
	**OR**
	PSB ≥10^3^ CFU/mm^3^
	**OR**
	BAL culture with ≥10^4^ CFU/mm^3^ within 2 days before or after the onset of a VAC, excluding the first 2 days of MV
	**IF NEGATIVE**
	Use of new antibiotics continued for at least 4 days within 2 days before or after the onset of a VAC, excluding the first 2 days of MV
Ventilator-associated pneumonia	VAT
	**PLUS**
	Presence of new or progressive pulmonary infiltrates

PEEP - positive end-expiratory pressure; FIO_2_ - fraction of inspired oxygen; VAC - ventilator-associated condition; ETA - endotracheal aspirate; PSB - protected specimen brush; BAL - bronchoalveolar-lavage; CFU - colony-forming units; MV - mechanical ventilation; VAT - ventilator-associated tracheobronchitis.

Unfortunately, recent developments in lung imaging in critical care patients have not been promising. We do have new tools, such as lung ultrasonography; however, the methodology to diagnose VAP has not been adequately validated. In addition, these techniques, while potentially easy to perform, are still associated with a steep learning curve. Ideally, portable CT scans will help in the future, but this technology is not available in most hospitals worldwide. When choosing to bring a patient to a CT unit, it is important to keep in mind the potential deleterious effects of transport in critically ill patients. For instance, Bercault et al conducted a study in 118 patients transported out of the intensive care unit (ICU) and found that the intrahospital transport of mechanically ventilated patients was associated with an increased rate of VAP (odds ratio [OR] 3.1; 95% confidence interval [95%CI] 1.4 - 6.7).^(8)^

With respect to etiology, VAT does not seem to differ from VAP. There are several observational studies that show that nonfermenting gram-negative bacteria are the most frequent cause of VAT, and they account for approximately two-thirds of VAP episodes.^(9,10)^ Some authors have also reported that multidrug resistant (MDR) pathogens are present in as many as one-third of VAT episodes.^(11,12)^

Currently, the treatment of VAT has not been fully supported by any guidelines, including the recently launched Infectious Diseases Society of America and American Thoracic Society guidelines for the treatment of hospital-acquired pneumonia.^(5)^ While there are no randomized control trials showing a benefit of VAT treatment, a good number of observational studies have shown the association of inadequate treatment or no-treatment for VAT and the subsequent development of VAP.^(4,13-17)^

A meta-analysis evaluating various strategies for the treatment of VAT found that the administration of systemic antimicrobials (with or without aerosolized antimicrobials) in patients with VAT was not associated with decreased mortality (OR 0.56; 95%CI 0.27 - 1.14). However, when other endpoints were assessed, most of the studies that provided relevant data noted that administration of antimicrobial agents in patients with VAT was associated with a decreased frequency of subsequent pneumonia and an increase in ventilator-free days. However, it remains unclear whether the length of ICU stay and the duration of MV could be shortened.^(18)^

### Biomarkers

Despite the limitations, some biomarkers, namely, C-reactive protein (CRP) and procalcitonin (PCT), can provide additional useful information for the clinical, laboratory and radiologic evaluation of a patient with suspected VA-LRTI.^(19,20)^

Studies by our group showed that on the day of confirmed VAP, both CRP and PCT levels could be useful.^(21,22)^ Recently, we showed that among patients with documented VA-LRTI, the levels of CRP and PCT were significantly higher in VAP patients than in VAT patients.^(23)^ Moreover, we found that CRP and PCT concentrations in confirmed VAT patients were 14mg/dL and 0,64ng/mL (median), respectively. According to a recently proposed biomarker algorithm established to promote antibiotic stewardship,^(24)^ CRP was below the infectious threshold level of 3mg/dL in 12% of VAT patients. However, according to the same algorithm, PCT was below the threshold (1ng/mL) in 58% of the VAT patients and was < 0.25ng/mL in 16% of these patients. As a result, in the assessment of an individual patient under MV, CRP seems to be more useful in VA-LRTI patients, since its concentration increases in both VAP and VAT. In contrast, PCT seems to particularly increase in severe forms of VA-LRTI, that is, VAP, making it not overly useful in the situation of VAT suspicion.

### Tracheobronchitis in special populations

Some patient subgroups should be considered separately, as some epidemiological, microbiological and therapeutic aspects are specific to these subgroups.

Ventilated chronic obstructive pulmonary disease (COPD) patients are at increased risk of nosocomial respiratory infections. Nosocomial tracheobronchitis in COPD patients is associated with a prolonged duration of MV and ICU LOS.^(25,26)^ The presence of bacteria in the lower airways of patients with COPD implies a breach of host defense mechanisms, initiating a vicious cycle of epithelial cell damage, impaired mucociliary clearance, mucus hypersecretion, increased submucosal vascular leakage, and inflammatory cell infiltration, thereby promoting further dysfunction in host defenses and bacterial adherence and growth.^(26)^ Additionally, COPD patients are at increased risk of infection with MDR gram-negative bacilli, such as *Pseudomonas aeruginosa*.^(27,28)^ The presence of these MDR pathogens could be associated with a poor prognosis.^(28)^ Interestingly, in recent years, patients with COPD, especially those who are critically ill and admitted to an ICU, are being increasingly recognized as belonging to a population at particular risk for Aspergillus. A recent report identified Aspergillus tracheobronchitis in 5% of critically ill COPD patients.^(29)^ Therefore, although Aspergillus tracheobronchitis is not common in critically ill COPD patients, in this subgroup of patients, early suspicion, diagnosis, and treatment could be lifesaving.

In immunocompromised critically ill patients, such as those with hematological malignancies, cancer therapy or transplantation recipients, considering fungal (including Aspergillus) or viral etiologies of tracheobronchitis is crucial, as early suspicion, diagnosis and treatment are the cornerstones of proper management.^(30)^

The respiratory tract of patients with cystic fibrosis is frequently colonized with multiple potential pathogens. The development of tracheobronchitis in these patients is not rare, and the need for wide-spectrum antibiotics is common. The presence of VAT in these patients requires the consideration of potential resistant gram-negative bacilli, such as *P. aeruginosa* and carbapenem-resistant strains of *Klebsiella pneumoniae*, and other *Enterobacteriaceae*, such as *Alcaligenes xylosoxidans, Acinetobacter* spp., *Stenotrophomonas maltophilia*, and *Burkholderia* spp. as potential pathogens. This makes the empirical antimicrobial choice complex, and previous microbiological data availability might be useful to improve the appropriateness of and individualize treatment.

Chronic critically ill patients are a specific population with an increasing prevalence in the ICU.^(31)^ Critical illness is associated with prolonged MV, increasing the risk for ventilator-associated respiratory infections. A number of factors predispose these patients to infection, including host defense impairment and prolonged exposure to high bacterial loads. This exposure can occur through the airway, and proper care of respiratory therapy devices is essential to minimize the risk for infection.^(32)^ Ventilator-associated respiratory infections generally involve highly resistant pathogens, and individualized therapeutic decisions are necessary to enhance the odds of appropriate empirical therapy. In these patients, adjunctive nebulized antimicrobials have potential as a rescue therapy strategy.

### Ventilator-associated tracheobronchitis as a quality metric

Intensive care units have always been at the center of national quality improvement programs, first by the Institute for Healthcare Improvement^(33)^ in the US and followed by many international initiatives. However, it seems that despite the initial increase in reporting, recent data suggests a new phenomenon: the "eradication of VAP".^(34)^ This has been ascribed to several factors, from the penalization of ICUs to discrepancies in diagnostic measures, lack of a gold standard for diagnosis, and poor agreement between clinical and surveillance methodologies, among others.^(1,35-37)^

In recent years, VAT has been progressively studied, and evidence has demonstrated its impact on the outcomes of ICU patients.^(4)^ The increase in VAT diagnoses in ICUs has had some unintended consequences. The first of which was that some patients who would have been previously diagnosed with VAP were now diagnosed with VAT. However, as VAP, like other potentially avoidable hospital-acquired infections, started to be monitored and in some cases associated with financial penalties, the incentive to misclassify VAP as VAT increased, as VAT was associated with minor or no financial consequences and did not potentially decrease the ICU's quality indicators.

For the above-described reasons, perhaps the best way to proceed would also be to include the VAT rate as a quality indicator. This proposition has evident pros and cons. Those in favor of it state that if the mechanisms of acquisition of VAT are similar to those of VAP and perhaps VAP is a continuum of VAT, then it is also a preventable condition.^(38)^ Additionally, as most ICUs diagnose and treat VAT, its impact on patient management, resource use and antibiotic use is not negligible.^(39)^ Finally, the application of this concept would adequately cover the full spectrum of "VA-LRTI", yielding realistic rates. Of course, there are limitations (Table 2) to this concept; the main one is the lack of robust evidence suggesting that the VAP bundle or specific VAP preventive measures are effective for VAT prevention.

**Table 2:** Limitations to ventilator-associated tracheobronchitis as a quality indicator

VAT surveillance definitions are subjective
VAT surveillance is very complex to automate
Discordance regarding the diagnosis of VAT is frequent
Mortality attributable to VAT is low
Little evidence of VAT preventive measures and its impact on outcomes exists
It is difficult to compare VAT rates between ICUs

VAT - ventilator-associated tracheobronchitis; ICU - intensive care unit.

Thus, future studies on VAP prevention should be extended to VAT to produce the necessary evidence. Moreover, ideally, indicators should be safe, effective, efficient, equitable, and timely. Therefore, it would be useful, perhaps, to create future initiatives to develop quality indicators that are less prone to interobserver variability (e.g., antibiotic dose after 48 hours in the ICU as a surrogate of ICU-acquired infections).^(40,41)^

## CONCLUSIONS

Ventilator-associated tracheobronchitis is a frequent and clinically relevant infectious complication in patients under mechanical ventilation for more than 48 hours, with a similar incidence to ventilator-associated pneumonia. Although associated with a significantly lower mortality than ventilator-associated pneumonia, tracheobronchitis survivors had a similar duration of mechanical ventilation and length of stay in the intensive care unit. Additionally, the progression of ventilator-associated tracheobronchitis to ventilator-associated pneumonia was significantly increased when patients with ventilator-associated tracheobronchitis were given inappropriate or no antibiotics. Although these disorders could occur independently, some evidence suggests that they are somewhat related and that, if left untreated or inappropriately treated, ventilator-associated tracheobronchitis is highly likely to progress to pneumonia.

Finally, we acknowledge the urgent need for a consensus in the diagnosis and management of ventilator-associated tracheobronchitis. Further large randomized studies are needed to clarify the effect of appropriate antibiotic treatment on the length of stay in the intensive care unit and duration of mechanical ventilation in these patients.
