# Erratum

**DOI:** 10.5935/0103-507X.20200027

**Published:** 2020

In the article Ventilator-associated tracheobronchitis: an update, with DOI number: 10.5935/0103-507X.20190079, published in the journal Revista Brasileira de Terapia Intensiva, 31(4):541-7, on page 541:

Where it read:

Nseir Saad

Read:

Saad Nseir

https://orcid.org/0000-0002-7618-0357

